# Genomic analysis of the original Elberg *Brucella melitensis* Rev.1 vaccine strain reveals insights into virulence attenuation

**DOI:** 10.1080/21505594.2018.1511677

**Published:** 2018-09-04

**Authors:** Mali Salmon-Divon, Adva Yeheskel, David Kornspan

**Affiliations:** a Genomic Bioinformatics Laboratory, Department of Molecular Biology, Ariel University, Ariel, Israel; b Bioinformatics Unit, George S. Wise Faculty of Life Sciences, Tel Aviv University, Tel Aviv, Israel; c Department of Bacteriology, Kimron Veterinary Institute, Bet Dagan, Israel

**Keywords:** Brucella *melitensis*, comparative genomic analysis, vaccine, virulence attenuation

## Abstract

The live attenuated *Brucella melitensis* Rev.1 Elberg-originated vaccine strain has been widely used to control brucellosis in small ruminants. However, despite extensive research, the molecular mechanisms underlying the attenuation of this strain are still unknown. In the current study, we conducted a comprehensive comparative analysis of the whole-genome sequence of Rev.1 against that of the virulent reference strain, *B. melitensis* 16M. This analysis revealed five regions of insertion and three regions of deletion within the Rev.1 genome, among which, one large region of insertion, comprising 3,951 bp, was detected in the Rev.1 genome. In addition, we found several missense mutations within important virulence-related genes, which may be used to determine the mechanism underlying virulence attenuation. Collectively, our findings provide new insights into the *Brucella* virulence mechanisms and, therefore, may serve as a basis for the rational design of new *Brucella* vaccines.

## Introduction

Brucellosis, also known as undulant fever or Malta fever, is the most common worldwide zoonotic bacterial disease, infecting over half a million people annually [1]. The cause of brucellosis is *Brucella* species: intracellular [2], Gram-negative bacteria, which were first isolated by Sir David Bruce (Malta, 1887) from the spleens of soldiers with fatal cases of the disease [1]. There are 10 known *Brucella* species, based on host specificity: *B. melitensis* (goats and sheep), *B. abortus* (cattle), *B. suis* (swine), *B. canis* (dogs), *B. ovis* (sheep and rams), *B. neotomae* (desert wood rats), *B. ceti* (cetacean), *B. pinnipedia* (seal), *B. microti* (voles), and *B. inopinata* (unknown) [1]. *B. abortus, B. melitensis*, and *B. suis* are the most pathogenic *Brucella* species for humans. The three *Brucella* species, *B. canis, B. ovis* and *B. neotomae* are of low pathogenicity for humans [1].

*Brucella* have a particular tropism toward the reproductive system of their primary animal hosts, which often leads to abortion in pregnant females and to sterility in males [1, 3]. Inside their hosts, *Brucella* reside within various types of cell, where they establish a replicative niche and remain protected from the immune response of their host [2]. *Brucella* can be passed on to humans upon direct contact with fluid discharges from an infected animal or by the consumption of dairy products made of unpasteurized milk, mainly goat milk and fresh soft cheese. Containment of human brucellosis depends upon the successful vaccination of livestock and imposing strict farming hygiene, surveillance, and infection control measures [1].

In the mid-1950s, Elberg and Herzberg developed a live attenuated *B. melitensis* vaccine strain, known as Rev.1, from the virulent *B. melitensis* 6056 strain (biovar 1). This strain was shown to successfully protect and reduce abortions in small ruminants [4], and it possesses several unique characteristics – including susceptibility to high concentrations of basic fuchsin and thionin (20 µg/ml), resistance to 2.5 µg/ml streptomycin, and susceptibility to 5 IU penicillin G [5] – factors that enable a clear distinction between the vaccine and virulent strains by using bacteriological tests. The original Rev.1 strain was later passaged by Elberg and, in 1970, passage 101 was made available as a freeze-dried seed stock culture. The strain originating from passage 101 resembles the original parental seed material, making it suitable for the prophylactic vaccination of sheep and goats [5].

To date, the attenuation mechanisms of the Rev.1 have not been fully characterized. Comparative genomic analysis of *Brucella* vaccine and virulent strains, had been widely used in order to reveal insights into virulence attenuation [6–9].A few comparative genomic and proteomic analyses of Rev.1 and the virulent reference strain 16M (biovar 1) have been conducted to elucidate the molecular mechanism underlying virulence attenuation [10–12]. These analyses revealed a panel of 32 genome-specific markers of the Rev.1 strain, as well as various differentially expressed proteins involved in iron metabolism, sugar transport, lipid metabolism, and protein synthesis. However, the genomic analyses were based on an assembled draft version of the Rev.1 genome, as there was no complete genomic sequence available at the time. In a recent study [13], we sequenced and annotated the whole genome of the original Elberg *B. melitensis* Rev.1 strain, passage 101, 1970. Using this information, we report, here, the first comprehensive comparative analysis of the complete genomic sequence of Rev.1 against the virulent strain 16M, aimed at elucidating the molecular mechanisms underlying the virulence attenuation of Rev.1. We have identified several candidate genes that could be related to the virulence attenuation of Rev.1 and may, therefore, facilitate the design of novel and better vaccines to control brucellosis.

## Results and discussion

### General genomic sequence features

We recently reported the complete genomic sequence of the original Elberg *B. melitensis* Rev.1 vaccine strain [13]. The estimated total genome size of this strain is 3,299,170 bp, and the genome comprises two large scaffolds, one of 2,121,368 bp and the other of 1,177,802 bp, which represent the two chromosomes of *B. melitensis* (chrI and chrII, respectively). The size of the Rev.1 chromosomes is highly similar to that of the 16M reference strain chromosomes (2,117,144 bp and 1,177,787 bp [14]). The detailed genome properties of the *B. melitensis* Rev.1 and 16M strains are listed in Table 1.

**Table 1.** Genomic features and properties of the newly sequenced genome of *B. melitensis* strain Rev.1, as compared with the known genome sequence of *B. melitensis* strain 16M.

	*B. melitensis* strain Rev.1		*B. melitensis* strain 16M	
Feature/Property	chrI	chrII	chrI	chrII
Size (bp)	2,121,368	1,177,802	2,117,144	1,177,787
GC (%)	57.15	57.34	57.2	57.3
Average gene length	826.89	878.79	850.5	905.34
ORF	2,044	1,067	2,032	1,067
tRNA	40	14	40	14
rRNA	6	3	6	3
Pseudogenes	99	82	115	88

**Figure 1.**
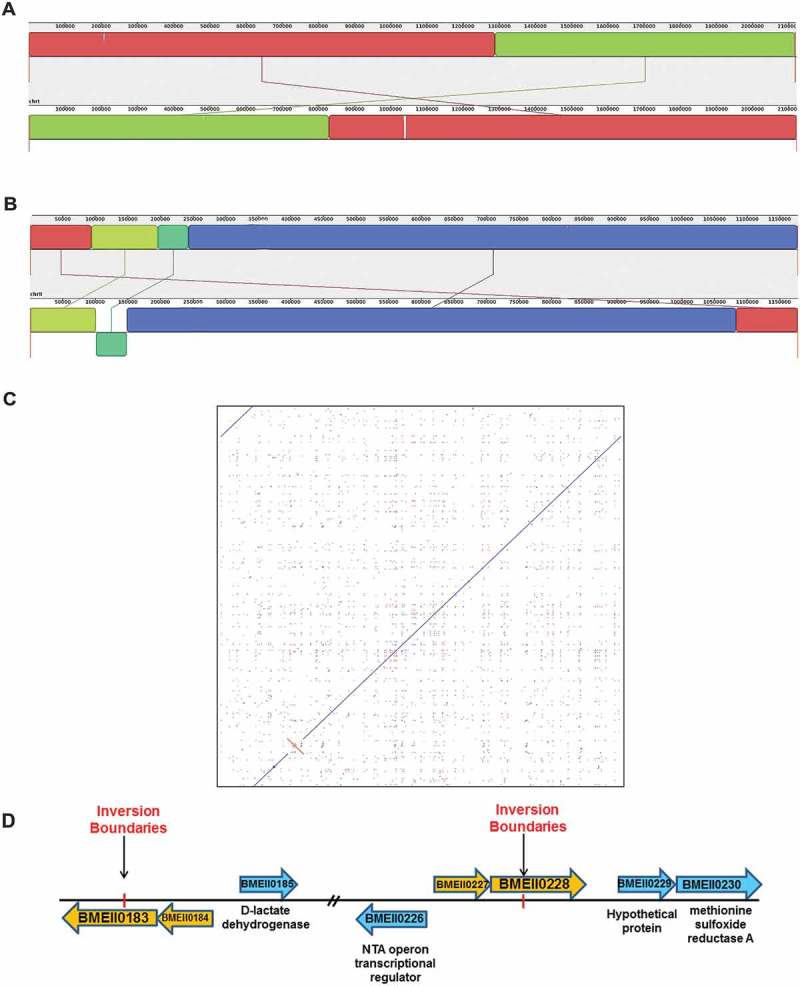
Whole-genome alignment of the *B. melitensis* 16M and Rev.1 strains. Mauve alignment of both chr I (**A**) and chr II (**B**) from the complete genomes of *B. melitensis* rev.1 (bottom panel) and 16M (top panel) are shown. The colored blocks indicate individual locally collinear blocks (LCBs). Same-colored blocks indicate homologous regions. The homologous LCBs are connected among the two strains. Same-colored blocks on opposite sides of the line indicate inversions. The boundaries of colored blocks indicate the breakpoints of genome rearrangement. (**C**) A dot plot comparing chr II of the Rev.1 and 16M strains using the LAST local alignment software (default parameters) [60]. The inversion found in the Rev.1 genome is indicated in red. (**D**) A scaled diagram of the ORFs located upstream and downstream of the inversion site on chrII of *B. melitensis.*

We conducted a genome-wide comparison between the Rev.1 and 16M strains to detect whole-genome similarities and regions of inversion, insertion, and deletion. The Rev.1 genome sequence shows an average nucleotide identity of 99.9% to the 16M genome, as well as a perfect genome-wide collinearity with the 16M strain (Figure 1(A,B)). Notably, chrII contains an inverted region of 46,874 bp (small green block in Figure 1(B)), which can clearly be seen on a dot plot generated by comparing chrII of the two strains (red alignment in Figure 1(C)). The inversion in chrII of Rev.1 (Figure 1(D)) occurred within two transposase elements (BMEII0183, BMEII0228) that are members of the IS3 family.

### Unique genomic regions

A comparative genomic analysis identified five regions of insertion (RIs) and three regions of deletion (RDs) within the Rev.1 genome, as compared with the 16M genome (Table 2).

**Table 2.** The RIs and RDs within the rev.1 genome.

Rev.1 RIs				Rev.1 RDs			
Name	Size(bp)	Genomic location	Gene ID	Name	Size(bp)	Genomic location	Gene ID
RI1	174	CP024715.1 (chrI): 1,639,618–1,639,792	BMEI1729	RD1	27	NC_003317.1 (chrI): 1,704,328–1,704,355	BMEI1651
RI2	135	CP024715.1 (chrI): 1,794,024 −1,794,159	BMEI1570	RD2	24	NC_003317.1 (chrI): 1,940,495–1,940,519	-
RI3	32	CP024715.1 (chrI): 38,246–38,278	BMEI1203	RD3	40	NC_003318.1 (chrII): 824,131–824,171	BMEII0784
RI4	3,951	CP024715.1 (chrI): 1,081,035–1,084,986	-				
RI5	24	CP024716.1 (chrII): 949,451–949,475	-				

#### Genomic insertions

**Figure 2.**
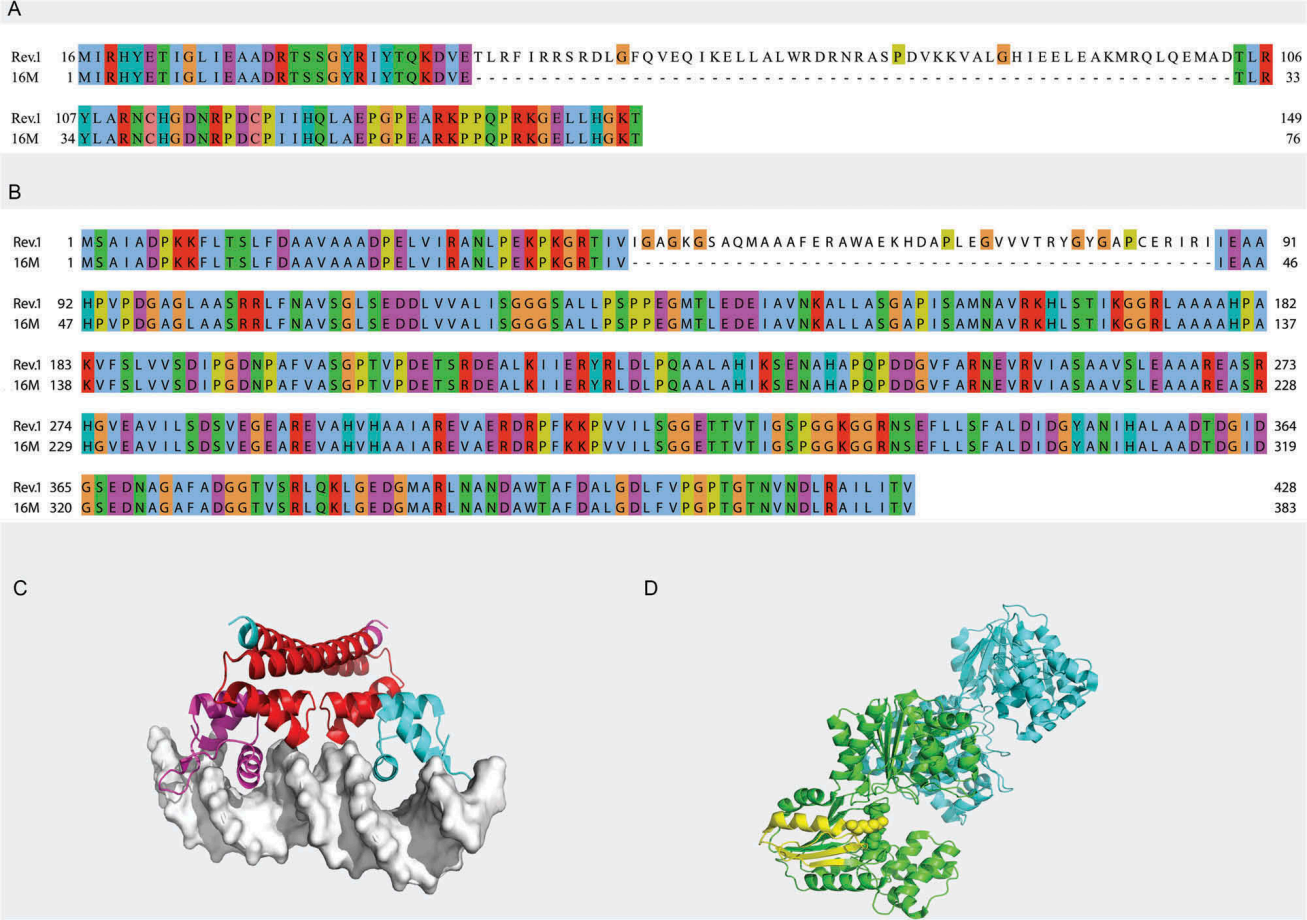
Regions of insertions within the MerR (**a**) and BMEI1570 (**b**) proteins of the *B. melitensis* Rev.1 strain. Pairwise alignment between the Rev.1 (top) and 16M (bottom) sequences was visualized using the Clustal X color scheme for residues coloring [63]. (**c**) Crystal structure of the *E. Coli* copper efflux regulator homodimer (4WLS). DNA is shown as white surface. The inserted sequence 1 (RI1) is shown in red. (**d**) Crystal structure of the *T. maritima* glycerate kinase (2B8N). The inserted sequence 2 (RI2) is shown in yellow. Active site residues are shown as spheres.

ChrI of Rev.1 contains three small RIs (174 bp, 135 bp, and 32 bp; RI1, RI2, and RI3, respectively) and one large RI (3,951 bp; RI4). A bioinformatic analysis revealed the location of RI1 within the ORF BMEI1729, which encodes the mercuric resistance operon regulatory protein, MerR, leading to the addition of 58 amino acids (Figure 2(A)). DNA-binding transcriptional regulators of the MerR family had previously been found in a wide range of bacterial genera and shown to respond to environmental stimuli, such as oxidative stress, heavy metals, or antibiotics [15]. As the MerR response to mercury(II) was previously shown to rely on the specific positioning and orientation of metal-binding amino acids within the protein [15], mutations in *merR* may play a role in bacterial attenuation.

RI2 is located within the ORF BMEI1570, which encodes a putative hydroxypyruvate reductase, leading to the addition of 45 amino acids to the N-terminus of the protein (Figure 2(B)). Hydroxypyruvate reductase metabolizes the toxic intermediate glyoxylate – a small and very reactive dicarboxylic acid molecule synthesized by most eukaryotes and prokaryotes [16]. Notably, the hydroxypyruvate reductase of the attenuated *B. melitensis* Δ*bpdA* phosphodiesterase mutant, which produces excess c-di-GMP, was shown to be downregulated, emphasizing the relevance of this enzyme in *Brucella* virulence [17].

Next, to evaluate the possible effect of RI1 and RI2 on protein functionality, we conducted a structural analysis of the amino acid sequences of the Rev.1 MerR and hydroxypyruvate reductase. The RI1 of MerR is located within two known domains: MerR (Pfam PF00376), positioned between residues 1–24, and the DNA-binding domain MerR-type helix-turn-helix domain (Prosite PS50937), positioned between residues 1–43. A homologue of MerR – the copper efflux regulator homodimer (PDB 4WLS) of *Escherichia coli* – was recently crystalized with DNA [18] and was shown to be a dimer comprising two N-terminal DNA binding domains connected by a dimerization helix [18]. Our analysis revealed that RI1 is located within the dimerization helix of MerR (Figure 2(C)); thus, the *B. melitensis* strain 16M, which lacks RI1, may possess a deficient dimerization of the DNA binding domain. RI2 of hydroxypyruvate reductase is located within a kinase domain (between residues 33 and 190), which, according to Pfam belongs to the DUF4147 family. The ORF BMEI1570 shares a 37% identity with the glycerate kinase of *Thermotoga maritima* (PDB 2B8N) [19]. The position of RI2, according to the crystal structure of the glycerate kinase of *T. maritima*, is indeed within its kinase domain (PDB 2B8N [19,Figure 2(D)). The inserted sequence folds into a helix and two β-strands, which are part of a 6-strand β-sheet, and contains one of the active-site residues. Notably, aligning 57 and 88 non-redundant protein sequences of the MerR and hydroxypyruvate reductase homologous (Supplementary Table S1A and S1B, respectively), revealed that most sequences contain RI1 and RI2, respectively (data not shown). We assume that RI1 and RI2 affect protein activity, although further studies are required to determine the possible association between these RIs and Rev.1 attenuation.

RI3 is located at the 3ʹ end of the ORF BMEI1203, which encodes ribonuclease D. An *in silico* analysis revealed an identical sequence immediately downstream of RI3 (data not shown); therefore, we assume that RI3 does not modify the translated protein. RI4 contains an ORF that encodes a transcriptional regulator belonging to the Cro/CI family. This region is inserted between a transposase element of the IS6 family (BMEI0200) and a hypothetical protein (BMEI0199). Transcriptional regulators, such as Cro/CI, regulate the expression of effector genes by binding to the promoter region, thereby positively or negatively affecting transcription to consequently modify the expressed mRNAs and the produced proteins [20]. A novel function of Cro/CI as a transcriptional regulator has recently been reported in *Staphylococcus aureus* [21], where the predominant clinical strain USA300 was shown to secrete reduced levels of virulence-associated proteins as a result of single-point mutations inside or immediately upstream the gene encoding Cro/CI [21]. Blat search analysis revealed homologs of the transcriptional regulator Cro/CI in other *Brucella* species, including *B. abortus* (strains 544, 2308, and S19), *B. suis* (strain 1330), *B. canis*, and *B. microti*. Further studies are required to determine the role of Cro/CI in *Brucella* transcriptional regulation and its possible association with bacterial attenuation. RI5 is located immediately downstream of BMEII0307, which encodes the WD40 repeat domain-containing protein, and does not affect the protein sequence.

#### Genomic deletions

We identified three RDs within the Rev.1 genome, as compared with the 16M genome (Table 2). ChrI of Rev.1 contains two small RDs of 27 bp and 24 bp (RD1 and RD2, respectively), while chrII contains one small RD of 40 bp (RD3). RD1 is located within the ORF BMEI1651, which encodes the urease accessory protein UreE1, leading to an in-frame deletion of His149-Gly157. Most members of the genus *Brucella* show strong urease activity [22], and it has been suggested that urease protects *Brucella* during their infection via the oral route, which is the major route of infection leading to human brucellosis [22]. Blat search analysis revealed a similar RD in *B. canis* and *B. suis* 1330. The importance of this RD in the attenuation of Rev.1 is questionable, as *ureE* in cluster 1 had previously been shown to be a pseudogene in *B. melitensis* and *B. suis* [23]. RD2 is located downstream of BMEI1888, which encodes lactoylglutathione lyase, and RD3 is located at the 5ʹ end of BMEII0784, which encodes a putative hydroxlase. Both BMEI1888 and BMEII0784 belong to the glyoxalase pathway, which plays an important role in detoxification by converting methylglyoxal into D-lactic acid [24]. The gene *glxI*, which encodes lactoglyglutathione lyase in *B. abortus* 2308, had previously been shown to be activated in macrophages 4 h post-infection [25], and its induction was suggested to contribute to *Brucella* proliferation and intracellular survival [25]. Our genomic comparative analysis revealed a similar RD3 in other *Brucella* species, including *B. abortus* (strains 544, 2308, and S19), *B. suis* (strain 1330), *B. canis*, and *B. microti*. Notably, we noticed a larger RD2, consisting of 40 bp, in the genome of *B. abortus* (strains 544, 2308, and S19); an insertion of 56 and 72 bp in the genomes of *B. canis* and *B. microti*, respectively; and a similar sequence to that of *B. melitensis* 16M in the *B. suis* (strain 1330) genome. As RD2 is divergent among *Brucella* species, we may consider it a potential target for the typing and detection of *Brucella*.

### Genomic SNPs

**Figure 3.**
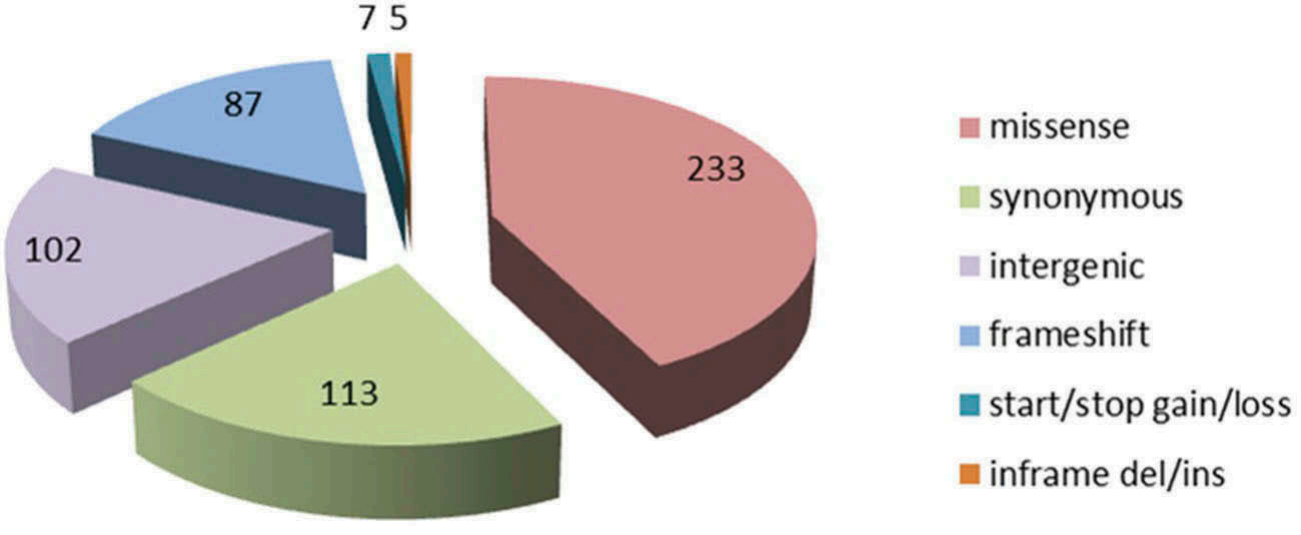
Distribution of functional Rev.1 SNP annotations, as detected by the SnpEff tool.

Genome-wide comparisons between Rev.1 and 16M were conducted to identify all the single-nucleotide polymorphisms (SNPs) between the two genomes. A total of 547 variants were detected (Supplementary Table S2A), representing a variant rate of 1:6,023 bp. Of these 547 SNPs, 102 SNPs (18.6%) are located in intergenic regions; 113 SNPs (20.6%) are synonymous substitutions encoding the same amino acid; 233 SNPs (42.5%) are missense substitutions encoding a different amino acid, and the remaining 99 SNPs (18%) contain mainly frameshift mutations, which, in many cases, have a deleterious effect on the protein function. The distribution of the various SNPs found in the assembled Rev.1 genome is presented in Figure 3. Genome-wide comparisons between Rev.1 and other virulent *B. melitensis* strains, including *B. melitensis* Ether, M28, ATCC 23,457, and NI revealed a total of 3,106, 2,581, 2,543 and 2,798 SNPs, respectively (data not shown). Our analysis revealed 109 variants that were similar in all five virulent strains (Supplementary Table S2B). *B. melitensis* isolates were previously shown to be clustered into five genotypes [26,27]. *B. melitensis* Ether was denoted as genotype I (Mediterranean strains); *B. melitensis* M28, ATCC 23457, and NI were denoted as genotype II (Asian strains); and *B. melitensis* 16M and Rev.1 were denoted as genotype V (American strains). The high number of SNPs detected between Rev.1 and the virulent strains Ether, M28, ATCC 23457 and NI indicate that these four virulent strains are indeed distant relatives of the vaccine strain, Rev.1. Thus, we next focused on the SNPs detected between Rev.1 and its closest relative, 16M.

To evaluate the SNP calling quality, we compared our set of 547 SNPs with those previously reported by Cloeckaert et al. [10]. and Issa et al. [11]. We found the previously described Rev.1 specific marker 272C> T mutation in the *rpsL* gene (BMEI0752) [10], and 31 of the 32 recently identified genome-specific Rev.1 markers [11]. We did not find the previously reported BMEI1592 442C> T SNP [11], although we noticed a similar SNP (403C> T) in a different location of the ORF BMEI1592. Blasting the BMEI1592 sequence against the Rev.1 draft genome available at NCBI (GenBank assembly accession: GCA_000158695.1) confirmed that the C > T SNP is indeed located at position 403 and not at position 442.

To detect novel mutations in Rev.1 virulence genes, we examined the list of *Brucella* virulence genes obtained from the *Brucella* Bioinformatics Portal [28,29] against our detected SNPs. Out of 212 reported *B. melitensis* virulence genes, we detected 18 potentially functional SNPs in the Rev.1 genome (Supplementary Table S3), which include the previously reported *rpsL* mutation (streptomycin resistance [10];) and 17 novel variations. Of these 18 variations, we validated five (Supplementary Table S3) using PCR followed by Sanger sequencing, so as to ensure the quality of our data.

Next, we compared our list of SNP-containing genes to a list of genes encoding for proteins that were previously reported to be under-expressed in Rev.1, as compared with 16M [12]. Out of the 25 reported under-expressed proteins, we detected five ORFs containing various SNPs (Table 3) that may be involved in the under-expression of these genes.

**Table 3.** List of ORFs that contain SNPs and encode reported under-expressed proteins in the *B. melitensis* strain Rev.1, as compared with strain 16M.

Locus tag	Definition	Mutation	Annotation
BMEI0010	chromosome partitioning protein parb	p.His277Arg	Missense variant
BMEI1923	isovaleryl-CoA dehydrogenase	p.Pro273Ser	Missense variant
BMEII0268	succinyl-diaminopimelate desuccinylase	p.Cys224Tyr	Missense variant
BMEII0435	D-ribose ABC transporter substrate-binding protein	p.Ala45fs	Frameshift variant
BMEII1048	60 kDa chaperonin groEL	p.Cys284Arg	Missense variant

### Virulence associated genes with differences between the Rev.1 and 16M strains

**Figure 4.**
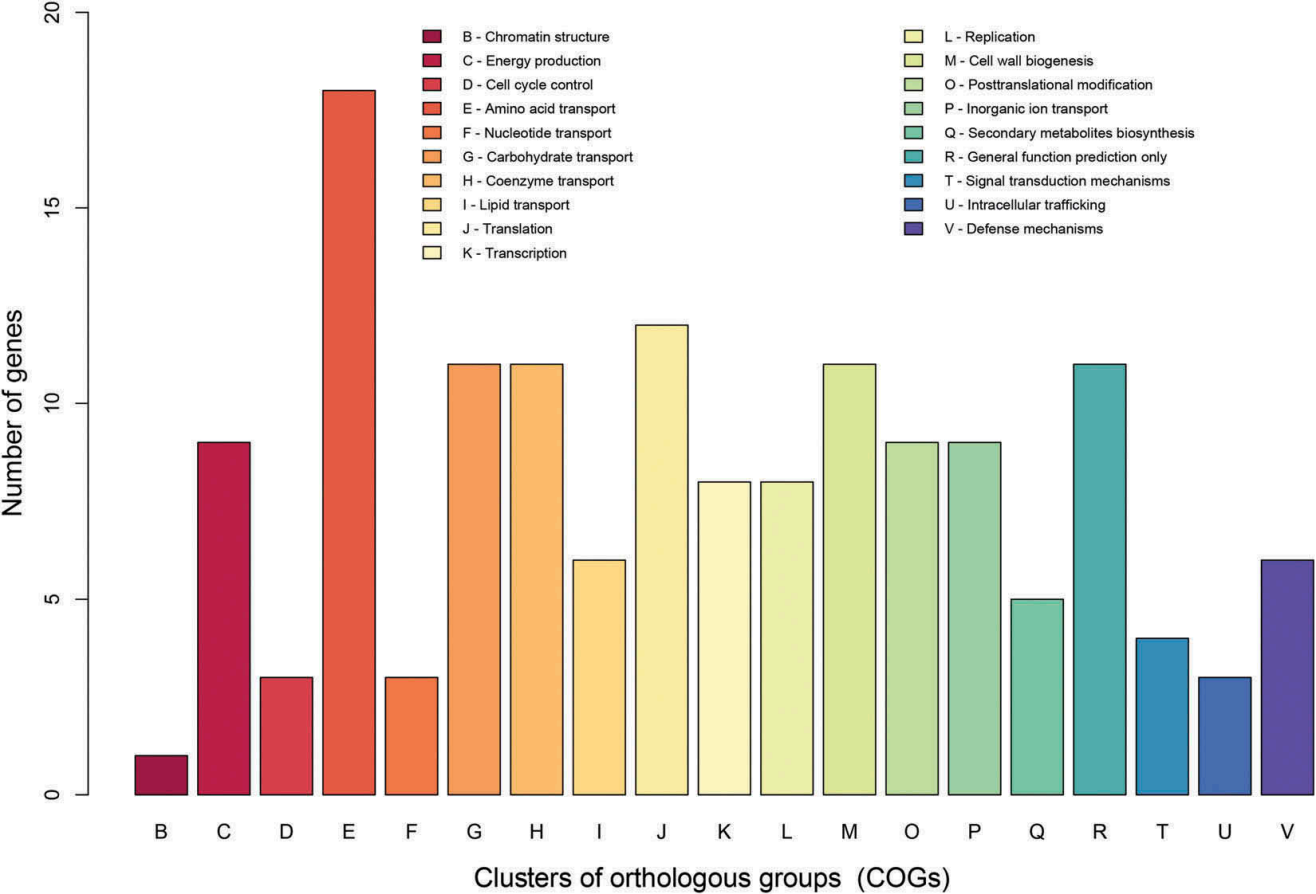
COG-based functional categories of the 160 ORFs that are different between the vaccine Rev.1 and the virulent 16M *B. melitensis* strains.

To identify genes that are potentially involved in the attenuation of Rev.1, we focused on the ORFs that are different between Rev.1 and 16M. Our SNP analysis revealed a total of 332 non-synonymous or frameshift mutations in 213 genes, of which 160 are protein-coding (Supplementary Table S4) and 53 are pseudogenes. The functional characteristics of the clusters of orthologous groups of proteins (COGs) of these 160 ORFs are shown in Figure 4. Enrichment statistical analysis revealed significant enrichment in the “defense mechanisms” COG term (Supplementary Table S5). In addition, we found non-synonymous or frameshift mutations in key genes encoding proteins that belong to the functional classes “lipid metabolism”, “stress proteins/chaperones”, “regulation”, “amino acid metabolism”, and “cell-wall synthesis”, as detailed below.

#### Lipid metabolism

The *Brucella* cell envelope contains the phospholipids phosphatidylcholine (PC) – whose synthesis has previously been shown to occur exclusively via the PC synthase (PCS) pathway [30] – and phosphatidylethanolamine [30]. We detected a missense mutation (c.86C> T) in the ORF BMEII0695 of Rev.1, which encodes PCS. The role of PCS in *Brucella* pathogenicity was reported by Comerci et al. [30], who showed that *B. abortus* possessing a *pcs* mutation display a reproducible virulence defect in mice. As the phospholipid composition of the membrane is critical for the interaction of *B. abortus* with the host cell [31], the Rev.1 *pcs* mutation may contribute to its attenuation.

#### Stress proteins/chaperones

Facultative intracellular *Brucella* are known to be capable of employing various strategies to survive the bactericidal environment within phagocytes and, thereby, to avoid the immune system of the host [32]. During this process, molecular chaperones, peroxiredoxins, and siderophores were shown to play an important role in *Brucella* survival [33–35]. Our SNP analysis revealed mutations in the ORFs BMEI2002 (c.25A> C; a missense mutation), BMEII1048 (c.850T> C; a missense mutation), and BMEI1619 (c.738T> A; a missense mutation), which encode the molecular chaperones DnaK, GroEL, and Hsp33, respectively. The induction of DnaK has been reported in *B. suis* [36], and the induced DnaK enabled *Brucella* to adapt to the hostile environment of the macrophage and was essential for the intracellular multiplication of *B. suis* [33,36]. We also found missense mutations in the ORFs BMEI1049 (c.167C> A) and BMEII0078 (c.485A> C), which encode a peroxiredoxin and the 2,3-dihydroxybenzoate-AMP ligase EntE, respectively. The *B. abortus* peroxiredoxin AhpC had previously been shown to detoxify endogenous H_2_O_2_ generated via aerobic metabolism [34]; the catecholic siderophores dihydroxybenzoate acid (DHBA) and brucebactin were shown to be produced and utilized as iron sources by *B. abortus* 2308 [35,37]; and 2,3-DHBA has been assumed to be required by *Brucella* at the state of gestation after changes in the iron status [35]. These data suggest that Rev.1 may have deficiencies in proteins that are crucially involved in stress tolerance.

#### Regulation

To survive extreme environmental changes and respond well to various stress conditions within their hosts, *Brucella* require a suitable regulation system [32]. Our SNP analysis revealed missense mutations in the ORF BMEI0685 (c.896C> G and c.884C> T), which encodes an AraC-like transcriptional regulator, and in the ORF BMEI1816 (c.443G> C), encoding the sensor histidine kinase RegB two-component system. The AraC-like transcriptional regulator DhbR was shown to regulate the biosynthesis of 2,3-DHBA and brucebactin, which play an important role in the virulence of *B. abortus* in its natural ruminant host [38]. RegA, the regulator of the RegB/RegA two-component system of *B. suis*, was shown to control oxidative respiration and denitrification, which enables *Brucella* to adapt to oxygen-limited conditions, produce sufficient energy under low oxygen conditions, and eliminate toxic NO produced by macrophages [39]. Thus, the regulatory capabilities of Rev.1 appear to be reduced, which may contribute to the attenuation of this strain.

#### Amino acid metabolism

Previous studies found that the replicative compartment of *Brucella* is poor in nutrients, such that the synthesis of amino acids is obligatory for its intracellular multiplication [40,41]. Indeed, in *B. suis*, mutations in genes that are involved in amino acid synthesis result in bacterial attenuation [40]. Our SNP analysis revealed missense or frameshift mutations in 18 genes involved in amino acid metabolism (Supplementary Table S5). Notably, we found that the ORF BMEI1759 possesses a missense mutation (c.523G> C) in the Rev.1 genome. This ORF encodes the vitamin B12-dependent methyltransferase MetH, which is involved in methionine biosynthesis and was previously screened in a murine infection model aimed at identifying pathogenic *Brucella* genes expressed *in vivo* [28,42]. Thus, we suggest that mutations in Rev.1 genes that encode enzymes involved in amino acid biosynthesis may contribute to the attenuation of this strain.

#### Cell-wall synthesis and penicillin resistance

The cell wall is the major shape-maintaining element in bacteria, and its integrity is crucial for cell viability. In both Gram-positive and -negative bacteria, the cell wall comprises a layer of the cross-linked polymer peptidoglycan (PG), which is composed of polysaccharides with alternating *N*-acetylglucosamine (GlcNAc) and *N-*acetylmuramic acid (MurNAc) saccharide groups. PG synthesis begins in the cytoplasm, where UDP-GlcNAc is synthesized from fructose-6-phosphate by the Glm enzymes, while UDP-*N*-acetylmuramyl-pentapeptide is synthesized from UDP-GlcN by the Mur enzymes (MurA, MurB, MurC, MurD, MurE and MurF) [43,44]. The proteins that catalyze the final steps of the PG synthesis include the bifunctional penicillin-binding proteins (PBPs), which catalyze the polymerization of the glycan strand (transglycosylation) and the cross-linking between glycan chains (transpeptidation). The penicillin-binding domain possesses a transpeptidase, a carboxypeptidase, or an endopeptidase activity. To destruct the PG layer of pathogenic bacteria, the PG synthesis pathway is often targeted, for example with β-lactam antibiotics. In contrast to 16M, Rev.1 is susceptible to 5IU penicillin G, thus allowing a clear distinction between virulent strains and the vaccine strain [5]. In *Streptococcus pneumoniae* and *Neisseria meningitidis*, the main mechanism of penicillin G resistance is the alteration of PBPs [45,46]. Point mutations in PBPs reduce the affinity for penicillin, which may increase penicillin resistance [47]. Additionally, penicillin resistance may be the consequence of penicillin acylase activity [48], which has been reported in many species of Gram-negative bacteria [49–51], leading to the hydrolysis of penicillin, with the production of the relatively inactive 6-aminopenicillanic acid. Another common mechanism underlying penicillin resistance is the activity of β-lactamase, which hydrolyzes the β-lactam ring and, consequently, deactivates β-lactam antibiotics [52].

Our comparative analysis of PBPs and penicillin acylases (ORFs BMEI0573, BMEI0914, BMEI1055, BMEI1351, BMEI1831, BMEII0253, BMEII0211, BMEII0212, BMEI0913, and BMEI0814) in the 16M and Rev.1 strains revealed a 100% identity in nucleotide and amino acid sequences (data not shown). Furthermore, we did not find homologs of genes encoding β-lactamase enzymes in the genomes of the two strains. However, our SNP analysis revealed three genes in the Rev.1 genome, encoding proteins involved in cell-wall synthesis, which may be associated with penicillin susceptibility of this strain: BMEI0574 (c.4G> A; a missense mutation), which encodes the UDP-N-acetylmuramoyl-L-alanyl-D-glutamate–2,6-diaminopimelate ligase MurE; BMEI1302 (c.968G> A; a missense mutation), which encodes soluble lytic murein transglycosylases; and BMEII0839 (c.421A> C; a missense mutation), which encodes the undecaprenyl-phosphate/decaprenyl-phosphate GlcNAc-1-phosphate transferase. MurE has been previously associated with resistance to β-lactam antibiotics in *S. aureus* [53], and a mechanism was suggested whereby the suppression of β-lactam antibiotic resistance in MurE mutants is related to the reduced cellular activity of PBP2A and PBP2 – the two main proteins involved in the β-lactam antibiotic resistance in *S. aureus* [53]. Recently, MurE has been implicated in the transformation of high β-lactam antibiotic resistance from *Streptococcus oralis* into *S. pneumoniae* [54].

Another important finding in our analysis is a missense mutation in the ORF BMEI0123 (c.33C> G), which encodes the peptidyl-prolyl cis-trans isomerase. This protein had previously been reported to be one of the four proteins uniquely expressed by the *B. melitensis* 16M strain, as compared with Rev.1 [12]. A recent study determined the role of PrsA – a peptidyl-prolyl cis-trans isomerase – in the folding of *Bacillus subtilis* PBPs [55]; in the absence of PrsA, PBP2a, PBP2b, PBP3, and PBP4 are unstable, which results in growth arrest. Furthermore, PrsA was shown to be involved in glycopeptide and oxacillin resistance in *S. aureus* [56], where the disruption of *prsA* dramatically decreases the resistance of methicillin-resistant *S. aureus* to oxacillin [56]. Our data suggest that impairments in one or more genes involved in PG synthesis may affect the penicillin susceptibility of Rev.1 and contribute to the attenuation of this strain.

## Conclusions

The widely used live attenuated *B. melitensis* Rev.1 vaccine strain successfully protects and reduces abortions in small ruminants [5], but the molecular mechanisms responsible for its attenuation remain unclear. Here, we performed a comparative genomic analysis between the whole-genome sequence of the original Elberg Rev.1 strain, passage 101, and the 16M virulent reference strain. Although the genomes of the two strains are 99.9% identical, we identified five RIs and three RDs in the Rev.1 genome, as compared with that of 16M. Two RIs are located within the ORFs BMEI1729 and BMEI1570, resulting in the addition of 58 and 45 amino acids, respectively. A structural analysis of the sequence of these proteins showed that these RIs are positioned in highly important locations within the proteins, namely, in the dimerization helix of MerR and in the kinase domain of hydroxypyruvate reductase, which presumably contributes to the activity of these proteins. The third RI, encoding a transcriptional regulator that belongs to the Cro/CI family and is absent in the 16M reference strain, is located between the ORFs BMEI0200 and BMEI0199.

A SNP analysis revealed non-synonymous and frameshift mutations in important virulence-related genes involved in lipid metabolism, stress response, regulation, amino acid metabolism, and cell-wall synthesis. We suggest that these genes are involved in the molecular mechanisms underlying Rev.1 attenuation, although conclusively determining their roles in the virulence attenuation of *B. melitensis* requires further characterization, for example, through mutation, complementation, transcriptomic, and host-response studies.

## Materials and methods

### Genomic data

Rev.1 and 16M genomes and annotations were downloaded from NCBI (accession numbers: NC_003317.1 and NC_003318.1 for 16M; CP024715 and CP024716 for Rev.1).

### COG analysis

Reverse position-specific BLAST (RPS-BLAST) [57] was used to search protein sequences against a local COG BLAST database, which was downloaded from NCBI. The expectation value (E) threshold was set to 0.01, and the BLAST output was parsed using an updated version of the cdd2cog.pl script [https://github.com/aleimba/bac-genomics-scripts/tree/master/cdd2cog] to obtain the assignment statistics of the COGs. COG assignment was performed to selected protein sequences (16M proteins whose corresponding Rev.1 genes carry a potentially functional mutation) and to the full list of the 16M annotated proteins. The enrichment p-value was calculated using the hypergeometric test, in which we set the population size to be the number of proteins out of all annotated proteins that had at least one COG assignment. Similarly, the sample size was the number of proteins with functional mutations that have COG assignments.

### Comparative genomic analysis

The average nucleotide identity (ANI) between the Rev.1 and 16M genomes was calculated using the ANI calculator (http://enve-omics.ce.gatech.edu/ani/). The whole-genome alignment between 16M and Rev.1 strains was conducted using the progressive Mauve algorithm included in the Mauve genome alignment package [58]. The Rev.1 genome was reversed and complemented before being subjected to the software. The locations of conserved and unique regions were retrieved from the backbone output file. Deletions and insertions were validated by retrieving the indel area together with flanking regions from each side, and these regions were aligned, using the blat software [59], against the relevant genome, following a correction of the indel borders. A dot plot was generated using the last-dotplot tool within the LAST local alignment package [60]. Pairwise alignment between the Rev.1 and 16M sequences was conducted using MUSCLE software [61] and visualized with Jalview [62]. Residues are colored according to the Clustal X color scheme [63].

### SNP detection

The MUMmer [64] SNP detection pipeline was used to detect SNPs. Briefly, the Rev.1 assembly was aligned to the 16M strain genome using the NUCmer program. The SNPs contained in the generated delta-encoded alignment file were fetched and reported by the show-snp utility. Next, output SNPs were converted to a VCF format using an in-house script and were subjected to annotation by the SnpEff tool [65]. The SnpEff database of the 16M genome (2002 assembly) was built based on a GenBank file that was downloaded from NCBI.

### Structural analysis

The Pfam [66] and Prosite [67] databases were used to obtain known functional domains of the protein sequence. The protein sequences for alignment were obtained from the universal protein knowledgebase, UniProt. Sequence alignments were viewed using Jalview [62]. Proteins with structural data from these families were used to inspect the 3D location of the inserted sequences and to predict their functional effect. 3D structures were viewed in PyMOL (The PyMOL Molecular Graphics System, Version 2.0 Schrödinger, LLC).

## Supplementary Material

The supplementary data for this article can be accessed here.
