# Supplementary material for: Genomic analysis of the original Elberg Brucella melitensis Rev.1 vaccine strain reveals insights into virulence attenuation
Source: Virulence. 2018 Sep 4;9(1):1436–48. doi: 10.1080/21505594.2018.1511677 (PMC6141144; doi:10.1080/21505594.2018.1511677)
Supplement: Supplemental Material [file kvir-09-01-1511677-s001.zip › S1A.docx]

**Supplementary Table S1A. List of microorganisms used to align protein sequences of MerR**

1. *Brucella abortus str. 2308 A*
2. *Ochrobactrum sp. EGD-AQ16*
3. *Ochrobactrum sp. P6BS-III*
4. *Ochrobactrum anthropi*
5. *Ochrobactrum grignonense*
6. *Ochrobactrum thiophenivorans*
7. *Rhizobiales bacterium 63-22*
8. *Phyllobacterium sp. Tri-48*
9. *Paramesorhizobium deserti*
10. *Bordetella genomosp. 10*
11. *Bordetella genomosp. 5*
12. *Achromobacter spanius*
13. *Devosia sp. 17-2-E-8*
14. *Devosia sp. H5989*
15. *Bosea vaviloviae*
16. *Agrobacterium genomosp. 5 str. CFBP 6626*
17. *Agrobacterium genomosp. 13 str. CFBP 6927*
18. *Agrobacterium genomosp. 1 str. S56*
19. *Agrobacterium rhizogenes*
20. *Rhizobium nepotum 39/7*
21. *Rhizobium sp. UR51a*
22. *Rhizobium radiobacter*
23. *Agrobacterium radiobacter DSM 30147*
24. *Devosia sp. LC5*
25. *Devosia riboflavina*
26. *Devosia soli*
27. *Microvirga ossetica*
28. *Salinisphaera sp.*
29. *Leptospira fainei serovar Hurstbridge str. BUT*
30. *Massilia sp. CF038*
31. *Massilia sp. Leaf139*
32. *Solimonas aquatica*
33. *Nitratireductor basaltis*
34. *Defluviimonas alba*
35. *Aureimonas phyllosphaerae*
36. *Aureimonas sp. Leaf454*
37. *Vulgatibacter incomptus*
38. *Erythrobacter sp. QSSC1-22B*
39. *Pseudomonas cedrina subsp. cedrina*
40. *Caballeronia glathei*
41. *Sphingomonas sp. MCT13*
42. *Sinorhizobium fredii (strain NBRC 101917 / NGR)*
43. *Massilia sp. WF1*
44. *Massilia sp. Leaf139*
45. *Pseudomonas geniculata N1*
46. *Stenotrophomonas maltophilia YH67_09220*
47. *Stenotrophomonas maltophilia SAMN04487782_2934*
48. *Kiloniella spongiae*
49. *Magnetospirillum sp. ME-1*
50. *Parvibaculum lavamentivorans (strain DS-1 / DS)*
51. *Burkholderia vietnamiensis (strain G4 / LMG 22)*
52. *Caballeronia mineralivorans PML1(12)*
53. *Pacificimonas flava*
54. *Uncultured marine microorganism HF4000_APKG1C9*
55. *Acinetobacter baumannii 1288284*
56. *Acinetobacter sp. 1564232*
57. *Mesorhizobium mediterraneum*
