# Supplementary material for: Genomic analysis of the original Elberg Brucella melitensis Rev.1 vaccine strain reveals insights into virulence attenuation
Source: Virulence. 2018 Sep 4;9(1):1436–48. doi: 10.1080/21505594.2018.1511677 (PMC6141144; doi:10.1080/21505594.2018.1511677)
Supplement: Supplemental Material [file kvir-09-01-1511677-s001.zip › S1B.docx]

**Supplementary Table S2A. List of microorganisms used to align the protein sequences of hydroxypyruvate reductase**

1. *Aquamicrobium defluvii*
2. *Ochrobactrum anthropi*
3. *Shinella sp. DD12*
4. *Rhizobium rhizogenes NBRC 13257*
5. *Rhizobium leguminosarum bv. phaseol*
6. *Nitratireductor basaltis*
7. *Mesorhizobium sp. ORS3359*
8. *Mesorhizobium sp. ORS3324*
9. *Sinorhizobium meliloti*
10. *Mesorhizobium sp. LSHC414A00*
11. *Mesorhizobium sp. LC103*
12. *Brucella ovis*
13. *Rhizobium sp. LC145*
14. *Rhizobium sp. Leaf453 ASG68_16230*
15. *Rhizobium sp. Leaf453 ASG68_26895*
16. *Aminobacter sp. Root100*
17. *Mesorhizobium sp. Root102*
18. *Rhizobium sp. Root1204 ASC96_11575*
19. *Rhizobium sp. Root1204 ASC96_16130*
20. *Rhizobium sp. Root1212*
21. *Rhizobium sp. Root483D2*
22. *Mesorhizobium sp. Root552*
23. *Mesorhizobium sp. Root554*
24. *Mesorhizobium sp. Root157*
25. *Mesorhizobium sp. Root172*
26. *Ensifer sp. Root278*
27. *Rhizobium sp. Root482*
28. *Paramesorhizobium deserti*
29. *Ensifer glycinis AU381_20770*
30. *Ensifer glycinis AU381_07310*
31. *Sinorhizobium saheli ATB98_10915*
32. *Sinorhizobium saheli ATB98_05780*
33. *Mesorhizobium sp. AA22*
34. *Ochrobactrum pseudogrignonense*
35. *Mesorhizobium sp. UASWS1009*
36. *Ensifer alkalisoli A8M32_24935*
37. *Ensifer alkalisoli GN=A8M32_13210*
38. *Mesorhizobium qingshengii*
39. *Mesorhizobium muleiense*
40. *Phyllobacterium sp. OV277*
41. *Phyllobacterium sp. YR620*
42. *Nitratireductor sp. ES.061*
43. *Paracoccus alcaliphilus*
44. *Salinihabitans flavidus*
45. *Mesorhizobium albiziae*
46. *Mesorhizobium sp. YR577*
47. *Sinorhizobium americanum CCGM7*
48. *Mesorhizobium sp. B7*
49. *Rhizobiales bacterium 65-79*
50. *Rhizobiales bacterium 63-7*
51. *Shinella sp. 65-6*
52. *Rhizobiales bacterium 63-22*
53. *Pelagibaca abyssi*
54. *Mesorhizobium sp. 65-26*
55. *Mesorhizobium sp. ORS3428*
56. *Ochrobactrum sp. P6BS-III*
57. *Pseudaminobacter manganicus*
58. *Mesorhizobium australicum*
59. *Rhizobium sp. R635*
60. *Ochrobactrum sp. A44*
61. *Sinorhizobium sp. CCBAU 05631*
62. *Ensifer sojae CCBAU 05684 SJ05684_c33200*
63. *Ensifer sojae CCBAU 05684 SJ05684_b44530*
64. *Sinorhizobium fredii CCBAU 83666*
65. *Ochrobactrum thiophenivorans*
66. *Mesorhizobium sp. WYCCWR 10195*
67. *Mesorhizobium mediterraneum*
68. *Mesorhizobium sp. BSA136*
69. *Rhizobium sp. FH14*
70. *Rhizobium fredii*
71. *Sinorhizobium sp. NG07B*
72. *Sinorhizobium sp. BJ1 CO676_13575*
73. *Sinorhizobium sp. BJ1 CO676_12640*
74. *Phyllobacterium sp. Tri-48*
75. *Sinorhizobium medicae*
76. *Sinorhizobium fredii*
77. *Sinorhizobium meliloti*
78. *Mesorhizobium amorphae CCNWGS0123*
79. *Rhizobium fredii*
80. *Mesorhizobium alhagi CCNWXJ12-2*
81. *Phyllobacterium sp. YR531*
82. *Mesorhizobium metallidurans STM 268*
83. *Rhizobium freirei PRF 81*
84. *Chelativorans sp.*
85. *Ochrobactrum intermedium 229E*
86. *Mesorhizobium sp. LSHC420B00*
87. *Ensifer adhaerens OV14*
88. *Mesorhizobium sp. LNHC220B00*
