# Supplementary material for: Genomic analysis of the original Elberg Brucella melitensis Rev.1 vaccine strain reveals insights into virulence attenuation
Source: Virulence. 2018 Sep 4;9(1):1436–48. doi: 10.1080/21505594.2018.1511677 (PMC6141144; doi:10.1080/21505594.2018.1511677)
Supplement: Supplemental Material [file kvir-09-01-1511677-s001.zip › S5.docx]

| **Supplementary Table S5.** COG-based functional categories of *B. melitensis* Rev.1 coding sequences that are different from 16M strain | | | |
| --- | --- | --- | --- |
| Functional Category | Number of ORFs (total, based on 16M annotation) | Number of non-identical ORFs | p value |
| [E] Amino acid transport and metabolism | 314 | 18 | 0.586 |
| [J] Translation, ribosomal structure and biogenesis | 164 | 12 | 0.25 |
| [G] Carbohydrate transport and metabolism | 178 | 11 | 0.47 |
| [R] General function prediction only | 320 | 11 | 0.98 |
| [H] Coenzyme transport and metabolism | 128 | 11 | 0.127 |
| [M] Cell wall/membrane/envelope biogenesis | 172 | 11 | 0.43 |
| [S] Function unknown | 268 | 10 | 0.96 |
| [C] Energy production and conversion | 168 | 9 | 0.66 |
| [O] Post-translational modification, protein turnover, and chaperones | 121 | 9 | 0.278 |
| [P] Inorganic ion transport and metabolism | 149 | 9 | 0.518 |
| [K] Transcription | 192 | 8 | 0.6 |
| [L] Replication, recombination and repair | 118 | 8 | 0.39 |
| [I] Lipid transport and metabolism | 106 | 6 | 0.6 |
| [V] Defense mechanisms | 35 | 6 | 0.014 |
| [Q] Secondary metabolites biosynthesis, transport, and catabolism | 59 | 5 | 0.26 |
| [T] Signal transduction mechanisms | 85 | 4 | 0.747 |
| [U] Intracellular trafficking, secretion, and vesicular transport | 51 | 3 | 0.58 |
| [F] Nucleotide transport and metabolism | 68 | 3 | 0.77 |
| [D] Cell cycle control, cell division, chromosome partitioning | 26 | 3 | 0.19 |
| [B] Chromatin structure and dynamics | 1 | 1 | 0 |
